# 23.4 MAPPING MAJOR MOLECULAR CHANGES IN THE DEVELOPMENT OF THE HUMAN CORTEX

**DOI:** 10.1093/schbul/sby014.094

**Published:** 2018-04-01

**Authors:** Maree Webster, Cynthia Shannon Weickert

**Affiliations:** 1 Stanley Medical Research Institute; 2 Neuroscience Research Australia: Schizophrenia Research Laboratory

## Abstract

**Background:**

The predominant neurodevelopmental theory of schizophrenia posits that there is a failure of normal synaptic loss believed to occur during normal adolescence. However, the most consistent neuropathology in the cortex of people with schizophrenia is a deficit in the γ–aminobutyric acid (GABA) inhibitory interneurons, not a reduction in presynaptic and postsynaptic elements. Thus, disruption to the normal development of cortical interneurons may lead to interneuron deficiency in schizophrenia. However, to understand if pathological changes in the brain of an adult with schizophrenia would be consistent with aberrant development, the known neuropathology must be placed in the context of normal human cortical development.

**Methods:**

We examined the molecular changes that occur in the synapses, interneurons and the neurotransmitter systems during normal development of the human prefrontal cortex of 68 brains from healthy individuals (1 month - 49 years).

**Results:**

Contrary to the prevailing view that synaptic pruning predominates during adolescent brain development, we found presynaptic mRNA and protein levels generally peak between 5–12 years of age and then remain stable through adolescence and into adulthood. Likewise, markers for dendritic spines peak in infancy and while mRNA levels then decline, protein levels are maintained throughout development. The various interneuron markers show three very distinct patterns of expression over development. Parvalbumin and cholecystokinin increase from infancy, whereas somatostatin, calretinin and neuropeptide Y decrease from infancy. Calbindin and vasoactive intestinal peptide peak in the toddlers and then decrease in adults in an inverted U shaped-pattern. Levels of mRNA for the GABA synthesizing enzymes GAD65 (GAD2) and GAD67 (GAD1) peak around 1 year of age and stay consistent through to adulthood. The postsynaptic GABAA receptor α1, β2 and γ subunits increase over the postnatal period to peak in adolescent/young adulthood whereas the α2 subunit shows the inverse pattern and decreases over the postnatal period. The dopamine receptor, D1, increase expression throughout the postnatal period to peak in early adulthood, whereas D2 and D5 show a continual decline throughout life. The NMDA receptors are highest in the first year of life and while subunits GRIN 2B, 2D and 3A all decrease throughout life, GRIN1 remains stable and GRIN 2A decreases after childhood. More recently data supporting the neuroinflammatory hypothesis of schizophrenia has merged with the neurodevelopmental hypothesis and posits that the strongest signal from GWAS studies in schizophrenia is in the C4 gene, which is a component of the complement cascade involved in normal synaptic development. We have initiated an examination of the various components of the complement cascade to determine if/when they are expressed in the human cortex and how they could be impacting the developing circuitry. Preliminary data shows C4 mRNA expressed at very low, but constant levels throughout postnatal life. In contrast, C3 mRNA is expressed at higher levels than C4, peaks in infancy and remains stable into adulthood. MAC protein (CD-59) mRNA which protects cells from complement mediated damage, is expressed at very low levels at birth but then increases significantly with age and is highest in adulthood, suggesting that significant changes in complement may occur after brain maturation is complete.

**Discussion:**

Together these findings show very dynamic and complex patterns of expression from birth to adulthood, with the most active growth phase and dynamic changes occurring in the early years before adolescence. Thus, an insult during these early years could profoundly affect the developmental trajectory.

